# Titanium Dioxide Nanotubes as Model Systems for Electrosorption Studies

**DOI:** 10.3390/nano8060404

**Published:** 2018-06-05

**Authors:** Xian Li, Samantha Pustulka, Scott Pedu, Thomas Close, Yuan Xue, Christiaan Richter, Patricia Taboada-Serrano

**Affiliations:** 1 Microsystems Engineering Ph.D. Program, Rochester Institute of Technology, Rochester, NY 14623-5603, USA; xl9206@rit.edu; 2 Department of Chemical Engineering, Rochester Institute of Technology, Rochester, NY 14623-5603, USA; spustulka3@gatech.edu (S.Pu.); spedu@andrew.cmu.edu (S.Pe.); tclose@mit.edu (T.C.); cpr@hi.is (C.R.); 3 Materials Science Program, University of Rochester, Rochester, NY 14627-0216, USA; yxue4@ur.rochester.edu; 4 Industrial Engineering, Mechanical Engineering and Computer Science, School of Engineering and Natural Sciences, University of Iceland, Reykjavik 600169-2039, Iceland

**Keywords:** electrosorption, titania nanotubes, nanostructured electrodes

## Abstract

Highly ordered titanium dioxide nanotubes (TiO_2_ NTs) were fabricated through anodization and tested for their applicability as model electrodes in electrosorption studies. The crystalline structure of the TiO_2_ NTs was changed without modifying the nanostructure of the surface. Electrosorption capacity, charging rate, and electrochemical active surface area of TiO_2_ NTs with two different crystalline structures, anatase and amorphous, were investigated via chronoamperometry, cyclic voltammetry, and electrochemical impedance spectroscopy. The highest electrosorption capacities and charging rates were obtained for the anatase TiO_2_ NTs, largely because anatase TiO_2_ has a reported higher electrical conductivity and a crystalline structure that can potentially accommodate small ions within. Both electrosorption capacity and charging rate for the ions studied in this work follow the order of Cs^+^ > Na^+^ > Li^+^, regardless of the crystalline structure of the TiO_2_ NTs. This order reflects the increasing size of the hydrated ion radii of these monovalent ions. Additionally, larger effective electrochemical active surface areas are required for larger ions and lower conductivities. These findings point towards the fact that smaller hydrated-ions experience less steric hindrance and a larger comparative electrostatic force, enabling them to be more effectively electrosorbed.

## 1. Introduction

The properties of charged interfaces and the electrosorption of ions on charged surfaces have a remarkable influence on the kinetics of various electrochemical processes, including those having technological importance such as water desalination and energy storage [1,2,3,4]. Electrosorption is a process where ions of opposite charge (counter-ions) are immobilized within a region known as the electrical double layer (EDL), which forms in the vicinity of the electrolyte-electrode interface when a low direct current potential is applied [5,6]. Electrosorption takes place during electrochemical reactions, charging/discharging of batteries, operation of electrochemical capacitors (ECs) and capacitive deionization (CDI) [7,8]. Recently, CDI technology, which relies on the reversible removal of ions from the solution by trapping them within the EDL, has gathered great interest due to its low energy cost, environmental friendliness, and no secondary pollution [9,10]. Electrochemical capacitors (ECs) use the electric field in the EDLs established at the electrode-electrolyte interface to store electrical energy. Without Faradaic (redox) reaction at EC electrodes, the EDL energy storage mechanism makes fast energy uptake and delivery, and good power performance possible [11,12]. Therefore, the properties of the EDL crucially determine the ions that can be removed from aqueous solutions in CDI and the energy that can be stored in ECs. Finally, the characteristics of the EDL directly influence the outcome of electrochemical reactions by adding another “resistance” or providing more “housing” for electrostatic charge storage to the processes. 

Previous literature on electrosorption mainly focused on electrode development, and was mostly for specific applications [13,14,15]. Porous carbons are often the choice of materials for electrosorption as they combine a high surface area and good electrical conductivity [16,17]. In spite of considerable efforts directed towards increasing surface area of carbon-based electrode materials in the last decades, CDI and ECs performance are still not at the required level in order to become competitive technologies. One of the factors contributing to this is the inaccessibility of the surface area and the disordered pore arrangements of carbon materials that result in only part of the total surface area being utilized during electrosorption. Additionally, it was found that electrosorption capacity is dependent on the type of ion and its characteristics, e.g., hydrated ion radius, charge and even specific interactions with the surface. These dependencies cannot be fully captured by the classical theory usually employed to model CDI and EC performance. Chen et al. noted that during CDI operation with activated carbon electrodes at the same concentration, smaller ions depicted size-affinity by being preferentially captured by EDLs [18]. Furthermore, they claimed that in a mixed Cl^−^ and NO_3_^−^ solution, Cl^−^ is preferably electrosorped over NO_3_^−^. This interesting observation suggests that specific interactions between the ion and electrode indeed played a role in the electrosorption process since the Cl^−^ and NO_3_^−^ have a similar hydrated radius (3.31 Å and 3.35 Å respectively), same concentration, and identical ionic charge in the test [18]. However, the current knowledge of this interaction is still rudimentary, and many experimental observations remain difficult to interpret because the property of carbon-based electrodes such as the architecture, composition, and pore size are difficult to control in order to perform a systematic study of these phenomena. 

A model electrode with tunable properties is required in order to better understand the effects of electrode properties and their interactions with different ions on electrosorption. Electrodes with defined and tunable architectures, where pore sizes and pore lengths can be well defined, would allow for systematic investigation of the effects of confinement and surface properties on the electrosorption of ions with specific characteristics [19,20]. Owing to well-defined pore diameters and lengths via anodization of titanium foil in specifically formulated chemical baths; highly ordered and self-organized titanium dioxide nanotubes (TiO_2_ NT) are a very appealing candidate for fulfilling this role [19,20]. A particular advantage of TiO_2_ NT is that the crystalline structure is easily tuned by simply annealing between 350–450 °C in air (from amorphous to anatase), without modifying the electrode architecture. Anatase TiO_2_ NT is a good approach to compensate for the low conductivity of amorphous TiO_2_ NT, while offering a potentially different ion-accommodation mechanism. Anatase TiO_2_ contains a cavity in its crystalline structure where a small ion could insert itself [21]. In the present work, TiO_2_ NTs were fabricated and tested for their suitability as model electrodes for electrosorption studies. Electrosorption of three monovalent alkaline cations (Li^+^, Na^+^, Cs^+^) was systematically explored in order to capture the interactive effects of the crystalline structure and hydrated ion radius on electrosorption capacity, charge dynamics and utilization of electrode surface area. The three ions chosen for the present work bear the same charge, but have distinctly different hydrated radiuses, which allowed us to investigate the effects of ion size during electrosorption. Additionally, the three ions selected are relevant in terms of their applications: desalination, energy storage and deionization of radioactive waste. The goal of the study was to demonstrate the suitability of anodized TiO_2_ NT electrodes to study how electrode properties and ion characteristics affect electrosorption.

## 2. Materials and Methods

### 2.1. Fabrication of Titanium Dioxide Nanotube Electrodes

Titanium dioxide nanotube electrodes (TiO_2_ NT) were fabricated following the method described in previous work [21,22]. Pieces of titanium foil (purity 99.7%, from STREM Chemicals Inc., Newburyport, MA, USA) were cut to the desired electrode size and rinsed with ethanol. A 150 mL solution of Ethylene Glycol and aqueous 0.05 mol/L ammonium fluoride with a volume ratio of 50:1 was introduced into a temperature-controlled anodization cell under dry air in order to synthesize electrodes at different anodization conditions. The foil was anodized at varying temperatures for 1 h in this solution with a platinum counter electrode at fixed voltages of 60 V. Temperature was one of the anodization parameters explored in order to define the electrode-surface architecture. Highly-ordered and uniform cylindrical tubes were the desired electrode architecture for the electrosorption studies. After anodization, the electrodes were removed from the solution, rinsed with methanol, and allowed to air-dry. Four electrode samples were prepared simultaneously in order to ensure consistent characteristics. Some of the samples were annealed in air at 425 °C for 1 h in order to change the TiO_2_ crystalline structure from amorphous to anatase—this structure has a higher reported electrical conductivity [19]. The mass of the NTs was determined prior and after annealing by weighing, in order to detect any change in mass that might have occurred during annealing. Scanning electron microscopy (SEM, Zeiss Auriga CrossBeam SEM) was used to characterize the morphology of TiO_2_-NT electrodes. The specific surface area was determined via statistical analysis of the dimensions of the nanotubes measured from SEM images taken at random locations on the electrodes synthesized for this work, in the same fashion as previous work [22]. The mass of the nanotubes was determined via direct weighing.

### 2.2. Electrochemical Tests

All electrochemical tests in this work were performed using a BASi Cell stand C3 potentiostat (Bioanalytical Systems BASi, West Lafayette, IN, USA), the counter electrode was a BASi MW-1032 platinum electrode, and the reference electrode was BASi Ag/AgCl (BASi MW-2052). The working electrodes were the TiO_2_ NT prepared as detailed above. Electrochemical tests were performed with three monovalent alkaline metal ions (Li^+^, Na^+^, Cs^+^) as counter-ions and the same co-ion (Cl^−^) in 20 mL of each of the following 0.1 M solutions: Sodium Chloride (NaCl), Lithium Chloride (LiCl), and Cesium Chloride (CsCl). The area of the working electrode was 2 cm^2^, corresponding to a mass of electrode (TiO_2_ nanotubes and titanium foil) equal to 0.010 ± 0.001 g in all cases. The Ag/AgCl reference electrode and the platinum-wire counter electrode were placed in the solution along with the working electrode in a typical three-electrode setup. The volume and concentration of the solutions were selected to ensure that ions were not depleted from the bulk solution at higher applied potentials. This was to avoid ion-concentration effects on electrosorption capacity. A blanking procedure was run before each electrochemical test, comprising a constant potential of +600 mV vs. Ag/AgCl being held for 5 min. This was done to ensure that each test had the same initial conditions. The potentiostat was programmed for each electrochemical test with the specifications described below. 

One chronoamperometry cycle (CA) included 2 s of quiet time with no applied potential (0 mV), 130 s at a constant negative applied potential (charge) and 130 s at the same constant positive applied potential (discharge). The pairs of negative and positive applied potentials during charge and discharge were −200 mV/+200 mV; −400 mV/+400 mV; and −600 mV/+600 mV. Each CA cycle (quiet time, charge, and discharge) was repeated at least fifteen times with a blanking procedure between each cycle in order to target true equilibrium electrosorption capacity with the measurements. 

In the electrochemical impedance spectroscopy (EIS) test, the three-electrode cell was set up in the same way as for the CA and CV tests. The EIS tests were conducted with a Gamry Reference 600 (Gamry Instruments, Warminster, PA, USA) with the amplitude of the sinusoidal AC voltage signal of 5 mV over the frequency range 1 mHz to 1 kHz. Electrochemical impedance spectroscopy (EIS) spectra were analyzed with the Z-view software. 

### 2.3. Electrosorption Capacity

Electrosorption capacity was calculated assuming that each electron unit of charge was neutralized by one ionic charge. The accumulated charge was calculated via numerical integration of the area underneath the current response curve with time during the charging cycle. Values for electrosorption capacity were double-checked via calculation of the charge released during the discharge part of the cycle.

## 3. Results

### 3.1. Surface Architecture of Anodized TiO_2_ Electrodes

**Figure 1:**
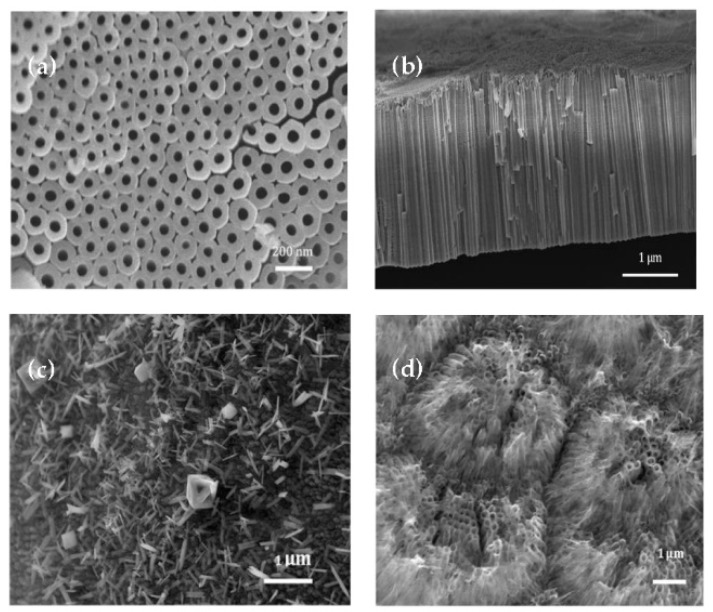
SEM images of TiO_2_ NT electrodes (**a**), top view of TiO_2_ NT anodized at 60 V 15 °C, annealed; (**b**) side view of TiO_2_ NT anodized at 60 V 15 °C, annealed; (**c**) top view of TiO_2_ NT anodized at 60 V 5 °C, non-annealed; (**d**) top view of TiO_2_ NT anodized at 60 V 35 °C, non-annealed).

Figure 1a,b shows SEM images (top and side view) of one of the annealed TiO_2_ NT electrodes anodized with a potential of 60 V for 1 h at 15 °C. Regular nanotube structure with uniform diameters and lengths were obtained at these anodization conditions, which were subsequently used in electrosorption experiments. Furthermore, the as-prepared TiO_2_ NT (non-annealed, amorphous) electrode is morphologically identical with the TiO_2_ NT after annealing at 425 °C for 1 h in air. Statistical analysis of SEM images was used in order to determine the mean diameter and length for the TiO_2_-NT electrodes prepared. Based on the measurement from the SEM images, it can be seen that the TiO_2_ NT electrode has a diameter of 41.4 ± 4 nm, length of 2136 ± 50 nm and specific surface area of 31.4 ± 2.2 m^2^/g. This level of control is highly desirable for electrosorption studies. However, it is also necessary to point out that surface architecture is highly sensitive to the anodization conditions. For instance, in Figure 1c, when anodization was carried out with the same anodization-bath formulation, applied potential and anodization time but at 5 °C, nano needle structures, instead of nanotubes, were obtained. This suggests that the low temperature may have changed the balance between the competitive oxidation reaction and oxide dissolution reaction taking place during Ti anodization in the presence of F^−^. Another example is provided in Figure 1d, in which the anodization conditions were the same, but the temperature was 35 °C. One can observe that the nanotube structure is less regular and defined than the one depicted in Figure 1a. This is because the high temperature accelerates both the fluoride etching and oxide layer growth, resulting in the cluster-like nanotube bulk appearance and deconstruction of some nanotubes. 

Electrodes of uniform TiO_2_ NTs were prepared at applied potentials equal to 60 V, a temperature of 15 °C, and an anodizaion time of 1 h for subsequent electrosorption studies, in order to demonstrate their applicability as model electrodes. Half of the electrodes were annealed in order to modify their crystalline structure without modifying their architecture. The annealed electrodes are identified as TiO_2_-NT-A, and the non-annealed electrodes are identified as TiO_2_-NT-NA hereafter.

### 3.2. Effects of Electrode Crystalline Structure and Ionic Strength on Electrosorption

**Figure 2:**
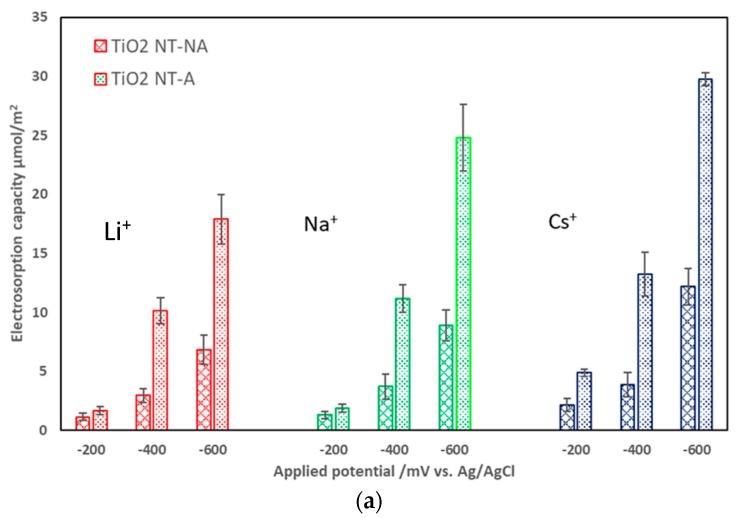

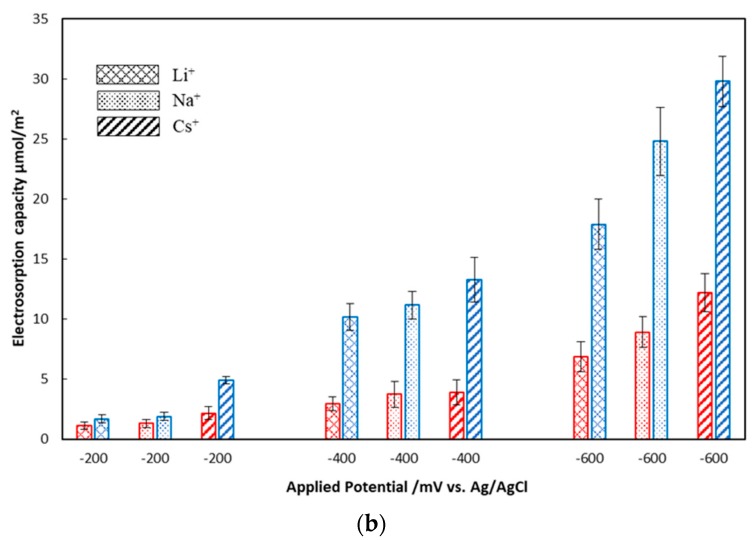
Electrosorption capacity of Li^+^, Na^+^, and Cs^+^ (**a**) at various applied potentials; and (**b**) for different electrodes (red bars for TiO_2_ NT-NA and blue bars for TiO_2_ NT-A).

The effect of the TiO_2_ NTs characteristic on electrosorption capacity was investigated in terms of its crystalline structure. As stated earlier, two groups of TiO_2_ NTs: amorphous (non-annealed, TiO_2_ NT-NA) and anatase (annealed, TiO_2_ NT-A) were selected in order to study the effect of the TiO_2_ crystalline structure on electrosorption capacity of similarly-charged ions. Anatase TiO_2_ NT electrodes are prepared via annealing the as-synthesized amorphous electrodes in air at 350 °C for 1 h. The surface structure (TiO_2_ NT diameter and length) of amorphous and anatase electrodes is identical, as it is not modified during annealing. Tighineanu et al. reported that the conductivity of anatase TiO_2_ can be higher than that of amorphous TiO_2_ by several orders of magnitude [19]. Due to high conductivity, it was expected that the TiO_2_ NT-A would lead to high charge densities at the surface of the electrodes, i.e., larger electrosorption capacities. Figure 2a,b depicts electrosorption capacity for Cs^+^, Na^+^ and Li^+^ for both TiO_2_ NT-NA and TiO_2_ NT-A electrodes. In Figure 2a, TiO_2_ NT-A electrode shows higher electrosorption capacity for each ion than that of the TiO_2_ NT-NA electrode, as expected. It is also shown that for TiO_2_ NT-A, the highest electrosorption capacity, 29.78 µmol/m^2^, was achieved during Cs^+^ electrosorption at −600 mV. As for TiO_2_ NT-NA, the highest electrosorption capacity was obtained for Cs^+^ at −600 mV with a value equal to 12.19 µmol/m^2^. This is only 40.9% of the highest electrosorption capacity of Cs^+^ on TiO_2_ NT-A at the same applied potential. Regardless of ion, the TiO_2_ NT-A electrode substantially exhibited better electrosorption performance, which can be attributed to the higher conductivity of TiO_2_-NT-A. Additionally, TiO_2_-NT-A possesses a tunnel structure of anatase that may potentially aid electron transport and ion accommodation. It should be pointed out that, at a specific applied potential, the electrosorption capacity for various ions with both TiO_2_ NT-A and TiO_2_ NT-NA electrodes follows the order of Li^+^ < Na^+^ < Cs^+^. For example, as seen in Figure 2b, with an applied potential of −600 mV, electrosorption capacities of Li^+^, Na^+^, and Cs^+^ with TiO_2_ NT-A were 17.88, 24.79, and 29.77 µmol/m^2^, respectively, while with TiO_2_ NT-NA, the electrosorption capacities were 6.82, 8.91, and 12.19 µmol/m^2^ for Li^+^, Na^+^, and Cs^+^, respectively. This trend can be explained by the fact that smaller hydrated ions experience less steric hindrance towards packing within the EDL and may also experience stronger electrostatic forces. In fact, the trend in electrosorption capacity aligns with the decreasing size of the hydration radii for Li^+^, Na^+^, and Cs^+^ (which are equal to 3.82 Å, 3.58 Å, and 3.29 Å, respectively) [20]. Furthermore, the comparative advantage of smaller hydrated ions towards electrosorption is more marked with TiO_2_ NT-A electrodes. For instance, at −200 mV, the elecrosorption capacity of Cs^+^ with TiO_2_ NT-NA was 2.9 times higher than that the corresponding value to Li^+^, whereas this value was equal to 6.1 in the case of TiO_2_ NT-A. The differences in electrosorption behavior did not limit themselves to capacity at equilibrium, but also in the dynamic behavior of the charging process (i.e., accumulation of charge within the EDL).

### 3.3. Charging Dynamics Dependence on Ion and Crystalline Structure

**Figure 3:**
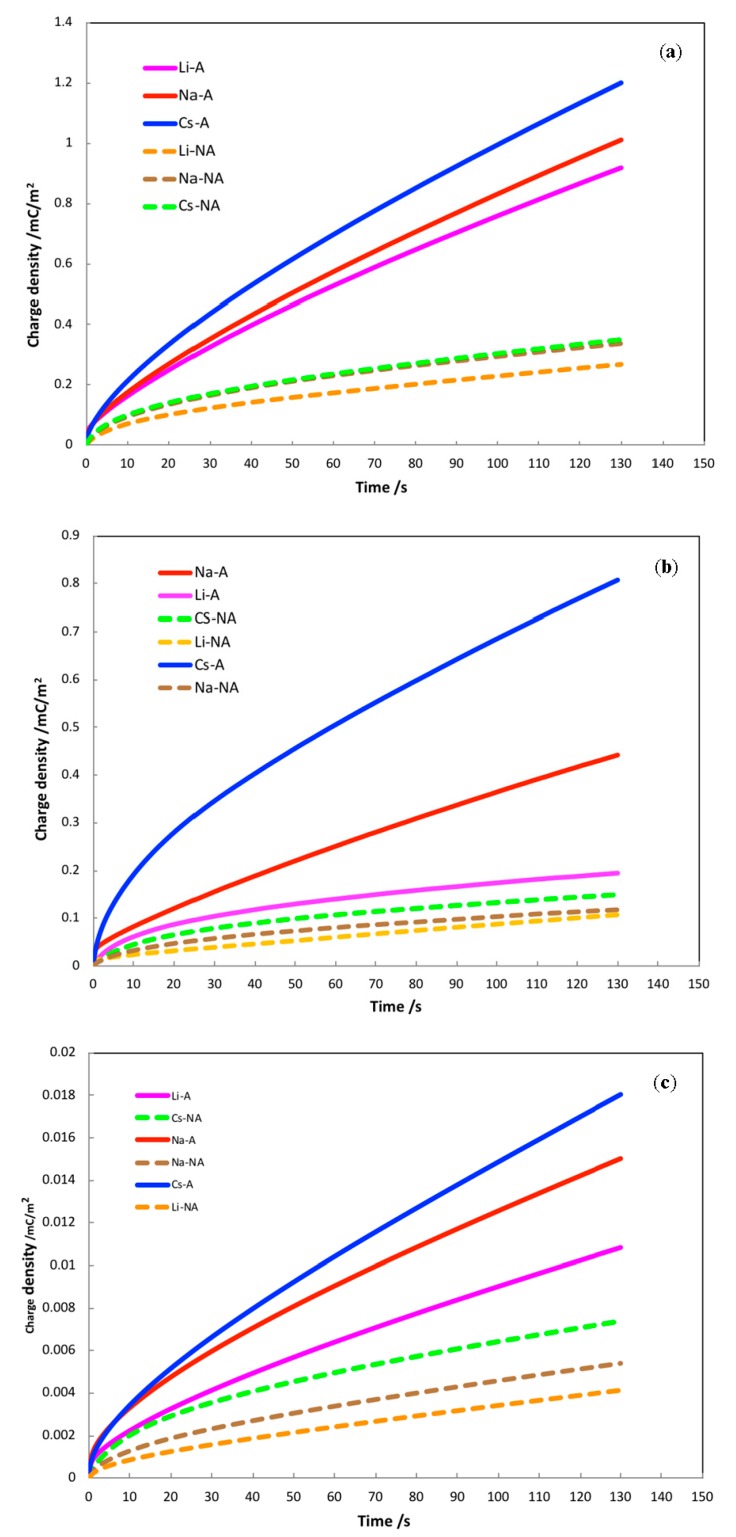
Charging rate of Li^+^, Na^+^, and Cs^+^ with TiO_2_-NT-A and TiO_2_-NT-NA under (**a**) −600 mV vs. Ag/AgCl; (**b**) −400 mV vs. Ag/AgCl; and (**c**) −200 mV vs. Ag/AgCl (solid line for TiO_2_-NT-A and dashed line for TiO_2_-NT-NA).

Figure 3a–c shows the dynamic behavior of EDL formation for TiO_2_ NT-A and TiO_2_ NT-NA under −200 mV, −400 mV, and −600 mV, respectively. When the applied potential was −200 mV, the highest charging rate was obtained with the TiO_2_ NT-A electrode and Cs^+^ in Figure 3a. Furthermore, it is evident that the charging rates for TiO_2_ NT-A were consistently higher than the rates obtained for TiO_2_ NT-NA with all the ions studied in this work. Increasing applied potential does not only increase the charging rate, but also accentuates the difference between charging rates of anatase and amorphous TiO_2_ NT electrodes. When the applied potential increased to −400 mV, it became more apparent of the advantage of TiO_2_ NT-A in terms of charging rate. From Figure 3b, two groups of charging curves are clearly identifiable resulting from the differences in the electrode crystalline structure. Figure 3c depicts even more dispersed behavior of the curves of charging rate, with a clear advantage for TiO_2_ NT-A. One should notice that the charging rate is consistent with the decreasing hydrated ion radii. As seen in Figure 3a–c, the charging rate followed the order of Cs^+^ > Na^+^ > Li^+^ with both TiO_2_ NT-A and TiO_2_ NT-NA. Interestingly, the dispersion among charging curves brought about by the differences in hydrated ion radii become more marked as applied potential increased. 

Summarizing, (i) TiO_2_ NT-A electrodes exhibited faster charging rates for each ion at every specific potential than that of the TiO_2_ NT-NA electrode due to the high conductivity of anatase; (ii) Cs^+^ with TiO_2_ NT-A consistently exhibited the highest charging rates at each tested potential, followed by Na^+^ and Li^+^. This last finding confirms the fact that smaller hydrated ions have the advantage of less steric hindrance and most likely stronger electrostatic interactions during electrosorption.

### 3.4. Electrochemically Active Surface Area

It is not difficult to conclude that ion electrosorption benefits from the large surface area of TiO_2_ NTs. The TiO_2_ NT-A and TiO_2_ NT-NA electrodes did not have any morphological difference, i.e., the surface structures on both electrodes were identical. Given the higher electrosorption capacity and charging rates exhibited by TiO_2_ NT-A, it was necessary to determine how much of the total surface area of TiO_2_ NTs was effectively used during electrosorption. Furthermore, it was necessary to explore how the TiO_2_ NTs crystalline structure and any possible electrode-ion interactions may have influenced the effective area involved in electrosorption. Electrochemical active surface area (EASA) for each case was estimated based on cyclic voltammetry (CV) and electrochemical impedance spectroscopy (EIS) measurements [23,24,25]. In order to obtain EDL capacitance via CV, a series of CV tests at multiple scan rates were carried out. The current in the non-Faradaic potential region, measured via CV, which is assumed to be generated due to double-layer charging exclusively, is equal to the product of the scan rate, v, and the double-layer capacitance, C_DL_, as given in Equation (1) [25,26].

(1) $$i_{\mathrm{DL}}{\;=\;\mathrm{vC}}_{\mathrm{DL}}$$

**Figure 4:**
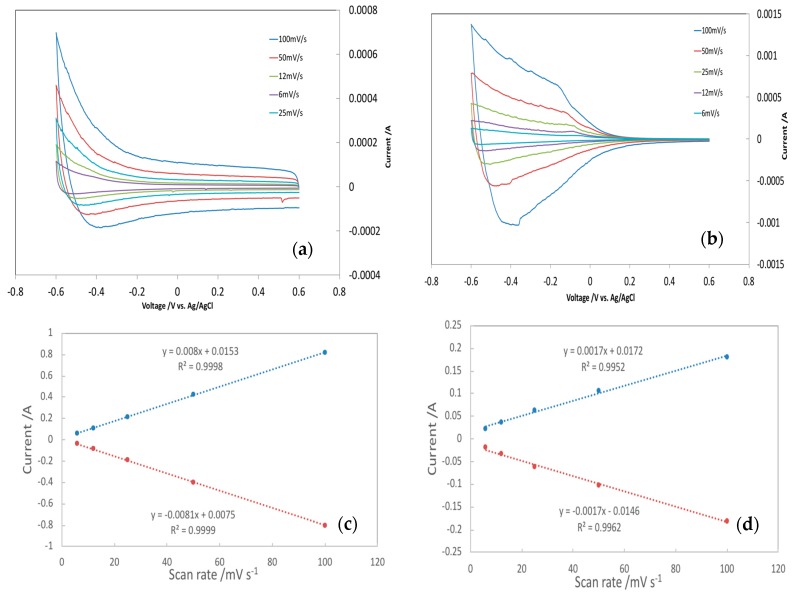
Cyclic voltammograms 1 M NaCl at various scan rates for (**a**) TiO_2_ NT-NA; (**b**) TiO_2_ NT-A; and EDL capacitance calculations for the determination of EASA (**c**) TiO_2_ NT-NA; (**d**) TiO_2_ NT-A. For the determination of EDL capacitance, charging currents measured at −0.3 V vs. Ag/AgCl, plotted as a function of the scan rate from Figure 4a,d, cathodic and anodic charging currents measured at −0.3 V vs. Ag/AgCl, plotted as a function of scan rate.

Examples of CVs of the TiO_2_ NT-NA and TiO_2_-A are depicted in Figure 4a,b. The CV tests were carried out in a non-Faradaic potential region at the following scan rates: 6, 12, 25, 50, and 100 mV/s. After acquiring the charging current at −0.3 V vs. Ag/AgCl, the cathodic and anodic charging currents were plotted as a function of scan rate v, which yields a straight line with a slope equal to C_DL_, as depicted in Figure 4c,d. The determined electrochemical double-layer capacitance of the system, C_DL_, is the average of the absolute value of the slope of the straight lines regressed from the experimental data.

Upon having the double-layer capacitance, C_DL_, the EASA of the sample was calculated according to Equation (2):

(2) $$\mathrm{EASA}\;=\;\frac{C_{\mathrm{DL}}}{C_{s}\;\cdot \;m}$$

where m is the mass of TiO_2_ NT, C_s_ is the specific capacitance of the sample and was calculated according to Equation (3):

(3) $$C_{s}\;=\;\frac{C}{A}\;=\;\frac{Q}{E\;\times \;A},\;\mathrm{where}\;Q\;=\;\overset{t}{\underset{0}{\int }}\mathrm{idt}$$

where i is the time-dependent current response in the CA test, t is the time of charge/discharge in the CA test, E is the applied potential in the CA test, Q is the total transferred charge in the CA test, and A is the area of the electrode.

**Figure 5:**
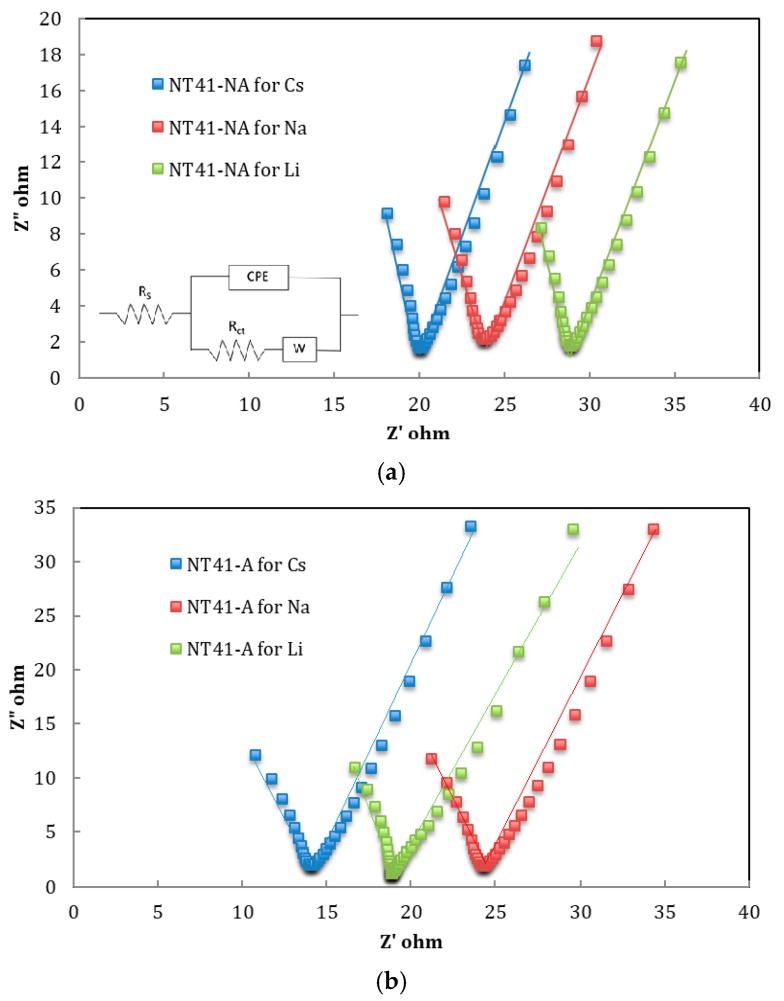
Nyquist plots for (**a**) TiO_2_ NT-NA and (**b**) TiO_2_ NT-A. The solid lines are the modeling fits to the EIS data by using the simplified Randles circuit shown in the inset of Figure 5a.

The double-layer capacitance was independently determined via electrochemical impedance spectroscopy (EIS). Figure 5a,b shows the Nyquist plots of different ions for TiO_2_ NT amorphous (NA) and anatase (A), respectively. The Nyquist plots are interpreted with the help of an equivalent circuit, shown in the inset of Figure 5, in which the electrochemical system is approximated by the modified Randles circuit. The intersection of the impedance spectra with the real axis at the high frequency region end is the bulk resistance (R_s_) including all contact resistance and resistance attributed to the electrolyte. The high frequency arc corresponds to the charge transfer limiting process and is ascribed to the charge transfer resistance (R_ct_) at the contact interface between the electrode and electrolyte solution in parallel with a constant phase element (CPE) related to the double-layer capacitance. The frequency-dependent impedance of the CPE is given by Equation (4) [27,28]:

(4) $$Z_{\mathrm{CPE}}\;=\;\frac{1}{Q_{0}\;\cdot \;{\left(i\omega \right)}^{\alpha }\;}$$

where Q_0_ is a constant with dimensions F s^−(1−^^α)^, ω is the frequency of the sinusoidal applied potential, i = (−1)^1/2^, and α is a dimensionless parameter, related to the phase angle of the frequency response, which has a value between 0 and 1 [27,28]. Based on the circuit model used here, the double-layer capacitance was calculated according to Equation (5) [27,28]:

(5) $$C_{\mathrm{DL}}{\;=\;Q}^{\frac{1}{\propto }}\;\cdot \;{\left[\left(\frac{1}{R_{s}}\;+\;\frac{1}{R_{\mathrm{ct}}}\right)\right]}^{\left(1\;-\;\frac{1}{\propto }\right)}$$

Note that when α = 1, the CPE behaves as a pure capacitor and C_DL_ = Q, and when α = 0, the CPE behaves as a pure resistor and C_DL_ is not detectable [25,26]. For example, from the EIS measurement of the Li^+^ with the annealed TiO_2_ NT electrode at an applied potential of −0.05 V vs. Ag/AgCl shown in Figure 5, R_s_ = 24.4 Ω, R_ct_ = 10.3 Ω, Q = 0. 148 mF s^−(1−^^α)^, and α = 0.605. The calculated C_DL_ from Equation (5) is 0.012 F. The calculated R_ct_ for all experimental conditions are listed in Table 1.

For a particular ion, the TiO_2_ NT-A results in a lower charge transfer resistance (R_ct_) than that of TiO_2_ NT-NA, as expected, due to the higher electrical conductivity of the anatase phase. Two components are expected to contribute to the charge transfer resistance in electrosorption. The first one is due to the process of ion diffusion from the bulk electrolyte to the electrode surface. The second contributing factor is due to entrapment of ions within the EDL. The experimental conditions chosen for this work ensured that the ion concentration in the bulk electrolyte is much higher than the equilibrium concentration of electrosorption so that the resistance due to diffusion can be, in principle, neglected. Therefore, the charge transfer resistance can be equated to the resistance that an ion at the surface of an electrode has to overcome in order to be immobilized within the EDL. The R_ct_ of different ions for both electrodes, TiO_2_ NT-A and TiO_2_ NT-NA, followed the trend of Li^+^ > Na^+^ > Cs^+^, which is the reversed order of the hydrated ion radius as seen in Table 1. This finding confirmed our previous conclusion that the smaller hydrated ion radii presented lower steric resistance and possibly stronger electrostatic interactions. In general, the EDL capacitance measured by EIS is within 10% of that measured by the scan-rate dependent CVs, and the EASA obtained for a given sample by the two methodologies tend to agree within ±10%. Values of EASA were also correlated to hydrated-ion radius, but with a reverse trend: the largest ion Li^+^ exhibits the largest EASA, followed by Na^+^ and then Cs^+^. It seems that packing of larger ions within the EDL requires more surface area. The EASA of 21.1 m^2^/g, which is found in the Li^+^ electrosorption test with TiO_2_ NT-NA, is the largest value reaching 67.1% of the total surface area, while the smallest EASA of 8.9 m^2^/g, 27.6% of the total surface area, is found in the Cs^+^ electrosorption test with TiO_2_ NT-A. 

Summarizing, anodized TiO_2_ NT electrodes of controlled surface structures proved to be a very effective model-electrode system in order to study experimentally ion-size effects and ion-electrode interaction effects during electrosorption.

## 4. Discussion

The crystalline structure of the TiO_2_-NT electrode was a determinant factor of electrosorption capacity and charging rate for all ions studied in this work. Due to the high conductivity, the electrosorption capacity of the anatase TiO_2_-NT electrode outperformed that of the amorphous TiO_2_-NT electrode for all three targeted ions, Li^+^, Na^+^, and Cs^+^ and tested potentials, −200 mV, −400 mV, and −600 mV. Additionally, the high conductivity of the anatase form of the TiO_2_-NTs is favorable for fast EDL formation. As the surface architecture for all electrodes is identical, the only difference between the annealed and non-annealed electrodes was in the crystalline structure. This mainly affects the electrical conductivity. However, a tunnel structure in the annealed (anatase) electrodes is not present in the non-annealed electrodes. This tunnel structure could potentially play a role as well.

The electrosorption tests evidenced that the hydrated ion radius plays a critical role during electrosorption. The electrosorption and charging rate of both anatase and amorphous TiO_2_-NT electrodes follow the same trend of Li^+^ < Na^+^ < Cs^+^, which agrees with the order of decreasing hydrated ion radius Li^+^ (3.82 Å) < Na^+^ (3.58 Å) < Cs^+^ (3.29 Å). The small ion can take advantage of low steric hindrance and higher relative electrostatic forces in order to be immobilized within the EDL more efficiently. This behavior becomes more marked with increasing applied potential. In fact, the lower charge transfer resistance is achieved by the combination of the anatase TiO_2_ electrode and the ion with the smaller hydrated radius.

Higher electrical conductivity translates into higher effective potentials at the solid-liquid interface of the electrodes, i.e., larger driving forces during EDL charging and formation. They also translate into larger surface charge densities to be neutralized via EDL, i.e., higher electrosorption capacities. If EDL charging and structure only responded to ionic strength (ion concentration and ionic charge), as predicted by Classical EDL Theory, no differences should have been detected for ions bearing the same charge at the same concentrations. This work confirms predictions of molecular modelling that EDL structure is determined by competitive energy and steric effects [29,30,31,32]. This fact can also be visualized via the determination of EASA through independent electrochemical techniques.

The EASA based on cyclic voltammetry (CV) and EIS agree with each other very well. The amorphous TiO_2_-NA electrode exhibited higher EASA than that of anatase TiO_2_-NT, which is contrary to the electrosorption capacity for any particular ion at the same potential. It is noted that the amorphous TiO_2_-NT utilized more surface area but reached a lower electrosorption capacity. The EASA of anatase TiO_2_-NT decreased with the ion hydrated radius, which indicated that smaller hydrated ions are more easily packed within the EDL, confirming the observations gained by measuring electrosorption capacity and charging rate. Furthermore, the lower EASA in the case of TiO_2_-NT-A confirms the fact that these electrodes present lower surface charge densities due to their lower electrical conductivity. The order of increasing EASA with increasing hydrated ion size (Cs^+^ > Na^+^ > Li^+^) further confirms the occurrence of steric hindrance in the structure of the EDL.

## 5. Conclusions

A series of highly-ordered TiO_2_ nanotubes (TiO_2_-NT) with two different crystalline structures, amorphous and anatase, were synthesized by anodization, and were used as model-system electrodes in the study of ion size and crystalline structure effects during electrosorption of alkaline metal ions. The electrodes of controlled geometry allowed for the systematic investigation of ion size and crystalline effects on electrosorption capacity, charging dynamics, and electrochemical active surface area (EASA), via well-known electrochemistry techniques. The simple geometry of the electrodes allowed for the clear identification of steric effects on the behavior of EDL formation and structure that had been predicted via molecular simulations, but had not been unequivocally determined via experimentation. The highly tunable, regular nanotube structure of the electrodes proposed in this work, made the elucidation of steric effects during electrosorption and EDL formation possible.

## Figures and Tables

**Table 1:** Electrochemically-active surface area (EASA) for TiO_2_ NT-A and TiO_2_ NT-NA determined via CV and EIS.

Electrode	Target Ion	R_ct_/Ω	EASA-CV/m^2^/g	EASA-EIS/m^2^/g	EASA-CV/Specific Area/%	EASA-EIS/Specific Area/%
TiO_2_ NT-A	Li^+^	10.3	14.1	13.7	44.7	43.6
TiO_2_ NT-NA	Li^+^	13.9	21.1	20.6	67.1	65.7
TiO_2_ NT-A	Na^+^	7.6	12.3	11.7	39.3	37.7
TiO_2_ NT-NA	Na^+^	9.9	17.8	16.8	56.8	57.7
TiO_2_ NT-A	Cs^+^	3.1	8.9	9.2	27.6	29.7
TiO_2_ NT-NA	Cs^+^	6.2	16.2	15.9	51.5	50.4
